# Bioprinting Scaffolds for Vascular Tissues and Tissue Vascularization

**DOI:** 10.3390/bioengineering8110178

**Published:** 2021-11-06

**Authors:** Peter Viktor Hauser, Hsiao-Min Chang, Masaki Nishikawa, Hiroshi Kimura, Norimoto Yanagawa, Morgan Hamon

**Affiliations:** 1 Department of Medicine, David Geffen School of Medicine, University of California Los Angeles, Los Angeles, CA 90095, USA; pvhauser@ucla.edu (P.V.H.); hsiaominchang@mednet.ucla.edu (H.-M.C.); nori@ucla.edu (N.Y.); 2 Medical and Research Services, Greater Los Angeles Veterans Affairs Healthcare System at Sepulveda, North Hills, CA 91343, USA; 3 Department of Chemical System Engineering, Graduate School of Engineering, University of Tokyo, Tokyo 113-8654, Japan; masaki@chemsys.t.u-tokyo.ac.jp; 4 Department of Mechanical Engineering, School of Engineering, Tokai University, Isehara 259-1207, Japan; hkimura@tokai-u.jp

**Keywords:** tissue engineering, vascularization, scaffold, scaffold-free, hydrogels, biomaterials, 3D printing, bioprinting

## Abstract

In recent years, tissue engineering has achieved significant advancements towards the repair of damaged tissues. Until this day, the vascularization of engineered tissues remains a challenge to the development of large-scale artificial tissue. Recent breakthroughs in biomaterials and three-dimensional (3D) printing have made it possible to manipulate two or more biomaterials with complementary mechanical and/or biological properties to create hybrid scaffolds that imitate natural tissues. Hydrogels have become essential biomaterials due to their tissue-like physical properties and their ability to include living cells and/or biological molecules. Furthermore, 3D printing, such as dispensing-based bioprinting, has progressed to the point where it can now be utilized to construct hybrid scaffolds with intricate structures. Current bioprinting approaches are still challenged by the need for the necessary biomimetic nano-resolution in combination with bioactive spatiotemporal signals. Moreover, the intricacies of multi-material bioprinting and hydrogel synthesis also pose a challenge to the construction of hybrid scaffolds. This manuscript presents a brief review of scaffold bioprinting to create vascularized tissues, covering the key features of vascular systems, scaffold-based bioprinting methods, and the materials and cell sources used. We will also present examples and discuss current limitations and potential future directions of the technology.

## 1. Introduction

Human organ function and physiology depend on a functional vascular system to facilitate oxygen and nutrient supply as well as the removal of metabolic products. Ischemia, a temporary or prolonged reduction in blood supply to a target organ, can lead to tissue dysfunction and necrosis [1] due to a lack of oxygen (hypoxia), nutrients, and a failure to eliminate metabolic waste products. In this context, avoiding ischemia time remains critical to preventing hypoxic injury and potential damages to transplant tissues and organs. Despite substantial progress achieved in creating three-dimensional (3D) blood vessels, fabricating a functional vascular multiscale system has remained a challenge that, consequently, hampered the ability of tissue engineers to create tissue constructs on the organ level. In addition, full vascularization of organoids is necessary for the complete development of organs, such as the kidney [2].

Many techniques have been developed to fabricate vascular networks that can mimic the complexity, the unique structures, and the functionality of human blood vessels [3]. Sheet rolling, tube molding, and direct scaffolding of biomaterials, with or without cells, are conventionally used to engineer vascular tubes [3]. However, these techniques have reduced control over the fabricated structure and are limited to the fabrication of large blood vessels. Recent advances in microfabrication and materials engineering [3] improved the control over the critical factors for the fabrication of in-vivo like blood vessels, including the cellular aspects (i.e., phenotypes [4], alignment [5], patterns [6]) and cell microenvironment [7,8]. Among these advancements, 3D bioprinting has become an essential tool for the fabrication of vascularized bioconstructs due to improved control over vascular growth, reproducibility, and scalability of the fabrication process [8]. In addition, the most recent innovations have integrated time-sensitive components into the bioprinting process, increasing the responsiveness level of the construct to environmental stimuli, improving the formation of realistic vascularized tissues [9,10]. In this review, we summarize the different methods, materials, and cell sources used for the 3D bioprinting of vascularization and vascularized tissues. We will then present relevant examples for the formation of vascularization networks in different engineered tissues and discuss the limitations and the future of the process.

## 2. Vascular System

To fabricate blood vessels and vascularized tissue in vitro, it is critical to understand the biological, physiological, and functional aspects of the in vivo system. The peripheral vascular system (PVS) includes all the blood vessels exiting the heart. Blood vessels are classified in arteries, capillaries, or veins. Arteries transport the blood from the heart to the periphery. They branch into smaller and smaller arteries until they branch into arterioles, metarterioles, and capillaries, the smallest and most numerous blood vessels. The primary function of capillaries is to accommodate the exchange of nutrients, gases, and bi-products between the blood and the cells. They form the connection between arteries and veins, which transport the blood back towards the heart. Capillaries connect to venules merging into increasingly larger veins. It is worth noting that large segments of the PVS are identified as separated anatomical structures while microvasculature (arterioles, metarterioles, capillaries, and venules) are parts of the tissue they vascularized [11,12].

**Figure 1:**
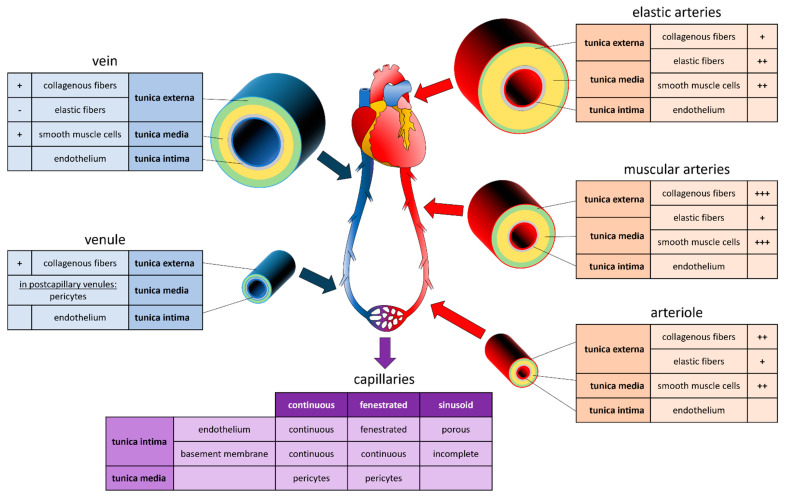
Composition of Vessels in the Cardiovascular System. Graphic and text inserts represent the composition of arteries (red), veins (blue) and capillaries (purple). For each section, the tunica externa (green), the tunica media (orange), and the tunica intima (grey) are indicated, as well as the proportion in collagenous fibers, elastic fibers, smooth muscle cells, and endothelium, ranging from lowest (-) to highest (+++) proportion.

The structure of the vessel varies according to the diameter of the vessels, which, in turn, is adapted to the hydrostatic pressure present in its interior (Figure 1). Aside from capillaries, blood vessels are composed of three concentric layers, starting from the lumen they are: the tunica intima, the tunica media, and the adventitia. The tunica intima is composed of a monolayer of endothelial cells (EC) that repose on a basement membrane composed of type IV collagen and laminin. The outer layer of the tunica intima is composed of an elastin layer, also known as the *internal elastic lamina*. The functions of the tunica intima are to contain cells and fluid within the vessel’s lumen and allow the blood to flow without problems. The tunica media is the middle layer of arteries and veins. It is primarily composed of smooth muscle cells (SMCs), collagen type I and type III, and variable amounts of elastic fibers, depending on the vessel thickness. It supports blood vessels and changes their diameter to regulate blood flow and blood pressure [13]. The external elastic lamina separates the tunica media from the adventitia. The outer layer, the adventitia, is composed of a loose connective tissue of type I collagen and fibroblasts. Its primary function is to restrain the vessel from excessive extension and recoil. These three layers are present in the macro- and micro-vasculature of arteries and veins, except for the capillaries where SMCs are absent and replaced by pericytes. The thickness of the layers is inversely correlated with the degree of branching.

Systemic arteries can be divided into two types, i.e., elastic and muscular, according to their tunica media’s relative composition in elastin and SMCs [14]. Arteries with close proximity to the heart, like the aorta and the pulmonary arteries, are very elastic. Their tunica media contains more elastin than SMCs. This characteristic allows them to maintain a constant pressure gradient, despite the periodic changes in cardiac flow volume. Muscular arteries are medium-sized arteries (e.g., femoral arteries, brachial arteries, radial arteries) and contain more SMCs than elastin in the tunica media. This allows them to contract and expand to adjust their diameter according to the peripheral blood demand, thereby controlling the blood flow in the capillaries. Capillaries are thin-wall vessels with lumen diameters of about 5 to 10 µm. Their thin wall, composed of a single endothelium layer, allows the exchange of nutrients, metabolites, and gases primarily via diffusion. In addition, differences observed in the capillary structure allow different solute permeability in different organs. Accordingly, capillaries are classified as continuous, fenestrated, and sinusoid capillaries [14]. Continuous capillaries have a continuous basement membrane, and their cells are connected by tight cell junctions. They are found in muscles, nervous systems, lungs, and skin [15]. Fenestrated capillaries receive their name from window-like transcellular openings, that generate a mesh-like structure. They are found in exocrine glands, renal glomeruli, and intestinal mucosa [15]. Finally, sinusoid capillaries have large interstitial gaps and an incomplete basement membrane. Sinusoid capillaries are found in the liver, spleen, and bone marrow [15]. Several capillaries join to form a venule. Venules have a diameter of 8 to 100 µm, with few SMCs, little elastin, and a thin adventitia. Venules join to form veins. Like arteries, veins are composed of three layers. However, veins are thin-walled, with a reduced tunica media and less elastic. This particularity allows the venous system to accommodate a large volume of blood (~3/4 of the circulating blood) at relatively low pressure. Unlike arteries, veins contain valves that prevent backflow and allow the blood to reach the heart [11].

## 3. Printing Methods

Tissue engineering aims to generate functional tissue by combining mechanical engineering, cell biology, robotic and material science. While generally, bioprinting aims to arrange cells in a 3D space according to the cellular and structural anatomy of a modeled tissue, different approaches have been developed to assemble cells to biosimilar tissues. For an overview of printing methods and their advantages and disadvantages, see Table 1. Unlike children’s’ building blocks, cells attach only in a limited fashion and commonly require adhesive components. The combination of cells and an adhesive or carrier material, usually a biopolymer, is called ‘bioink’. 

Pre-seeding and post-seeding are the two main strategies used to arrange cells in a bioprinted structure. In pre-seeding approaches, cells and the matrix are printed simultaneously, while in post-seeding, a bioprinted matrix is generated and subsequently populated with cells and cultured. Pre-seeding allows better control of the cellular component, as cell can be distributed more homogenous, and it seems to enhance cell retention [16]. On the other hand, post-seeding enables the use of printing conditions that are not conducive to cell viability (heat, UV) and gradual or staged seeding, allowing layering of different cell populations. The technical solutions also differ in how the cells are held in the physical space and cultured to maturation. This review introduces the principles of (i) sacrificial bioprinting and its different methods, (ii) the core/shell method, (iii) and 4D bioprinting.

### 3.1. Sacrificial

**Figure 2:**
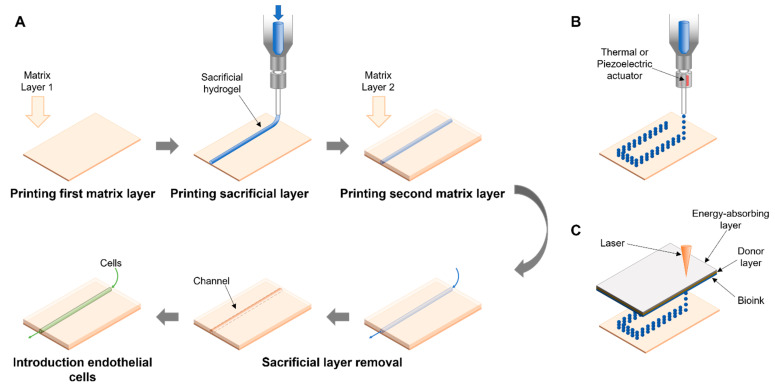
Principle of 3D Bioprinting Scaffold for Vascular Tissue Formation. (**A**) Sacrificial bioprinting. First step in sacrificial bioprinting is to deposit the sacrificial bioink or material onto a first layer of matrix. The sacrificial material (blue) is then covered with a second layer of matrix. Both matrices may be loaded with cells. After polymerization of the hydrogel, the sacrificial material is removed by chemical degradation, photolithography, temperature, or mechanical extraction, generating a hollow channel within the hydrogel block. In the last step, endothelial cells or endothelial progenitor cells are seeded into the hollow channel. (**B**) Droplet-based bioprinting. (**C**) Light- (Laser-)based bioprinting.

Sacrificial bioprinting is a technique in which a three-dimensional negative or hollow space is generated within a hydrogel or scaffold. The method is intuitively applicable to create a vascular network within a layer or block of engineered tissue [17]. In a first step, the sacrificial degradable hydrogel is deposited to generate a path for a vascular network that can supply the engineered tissue. After that, a hydrogel, or bioink, containing the cells of the future engineered tissue is used to cover the sacrificial deposit. After polymerization, the sacrificial hydrogel is removed by different methods, such as chemical degradation [18], photolithography [19], temperature [20] or mechanical extraction [21]. Finally, endothelial cells are seeded into the hollow channels from which the sacrificial material has been removed, generating a vascular network within the cells embedded in the hydrogel block (Figure 2).

The basic method of sacrificial bioprinting can be combined with various strategies to deposit the sacrificial ink or material. Depending on the method used to deposit the sacrificial material, bioprinting is categorized into extrusion based, droplet-based, laser-assisted, and core/shell-based. The advantages and disadvantages of the technology make them more or less suitable for certain applications. Strategies aiming to bioprint a vascular network need to be adapted to the particular vascular system that is to be generated. Techniques that are sufficient to generate simple vessels consisting of a single cell type or layer might require a different printing approach than major blood vessels consisting of several layers and cell types (see Figure 1).

#### Extrusion-Assisted Techniques

Extrusion-based bioprinting is the most common variant in 3D printing. In extrusion-based bioprinting, a liquid and often viscous solution is extruded, that is pushed from a syringe through a nozzle, and deposited according to the printing layout. Extrusion printing is an economical and versatile method that enables printing of bioinks with high-cell numbers quickly. The force to extrude the bioink through the nozzle is commonly generated pneumatically [22], via a piston [23], or a helical screwblade [24]. Extrusion printing can be divided into direct extrusion, where the bioink is delivered directly from the syringe’s nozzle to the print bed, and indirect extrusion, in which bioink is deposited in combination with supportive material. The challenge for both methods is to create a bioink, with a viscosity low enough to allow printing while rigid enough to hold the shape after printing and being biocompatible for cells to be viable [25]. Extrusion bioprinting is not ideally suited to print blood vessels, as it is challenging to lumen containing tubular structures with multiple layers.

Direct deposition of the bioink, or the sacrificial bioink on the printing bed is a straightforward approach but creating larger or complex structures can be difficult. To overcome the challenges of collapsing or deformed structures, the sacrificial bioink can be printed onto a printing bed or into a carrier matrix that suspends the printed structure. Noor and coworkers used a suspended direct printing approach to create perfusable cardiac patches and hearts [26]. For their personalized medicine approach, they isolated cells from patients’ omentum and reprogrammed and differentiated them into cardiomyocytes and endothelial cells. From the extracellular matrix (ECM) of the omentum they generated a hydrogel, which, combined with cardiomyocytes or endothelial cells, was used to formulate a bioink. Suspended direct extrusion printing was then used to generate vascularized cardiac patches or whole heart-like structures that could be transplanted back to the patient [26]. Extrusion bioprinting is only partially useful for printing vessels, as printing bifurcated vessels might be challenging. As mentioned above, the hydrogel carrier’s physical characteristics of the bioink are of great importance in extrusion-based bioprinting. In addition, the hydrogel should promote the differentiation or maturation of the printed cells.

Leucht et al. studied different modified-gelatin formulations to bioprint a bone and optimize the vascularization of the printed structure [27]. Gelatin can be generated with high viscosity and exhibit strong physical rigidity, but it cannot be crosslinked. Modification of gelatin can increase its suitability for bioprinting. Methacrylation, for example, can improve the printability by reducing viscosity, while acetylation can increase crosslinking. Leucht measured the physical properties of different gelatin, methacrylate-modified gelatin, and acetylated gelatin methacrylate formulations. They encapsulated human dermal microvascular endothelial and adipose-derived stem cells and then analyzed the vascularization of the printed bone. They found that a formulation combining methacrylated gelatin, gelatin, and acetylated gelatin methacrylate was best suitable for generating a vascular network and allowed differentiation of adipose-derived stem cells. Crosstalk between endothelial cells and adipose-derived stem cells promoted the osteogenic differentiation of adipose-derived stem cells [27].

Freeman et al. developed a 3D rotary direct extrusion bioprinter with the purpose of printing vessels of small diameter [28]. Instead of a printing bed, they used a polystyrene rod with a diameter of 4.9 mm that rotated along its rolling axis. On the surface of the rod, they applied a helical layer of bioink, composed of low passage primary neonatal human dermal fibroblasts mixed with fibrinogen and gelatin type A. After printing, the rod was submerged in thrombin to crosslink the fibrinogen and subsequently cultured them for up to 45 days, during which the fibroblasts differentiated and changed their phenotype into a spindle shape. Removal of the rods was followed by a significant reduction of gel length and luminal diameter. On day 7, the thickness of the vessel was ~300 µm when printed with 1 × 10^6^ cells/mL and <125 µm when printed with 3 × 10^6^ cells/mL [28].

Indirect extrusion printing is a sacrificial bioprinting method in which a sacrificial structure is fabricated to support the generation of larger tissues or scaffolds and later removed. It enables the production of complex tissues and further can be adapted for pre- and post-seeding strategies. Recently, indirect extrusion bioprinting has been demonstrated for nerve tissue applications [29], the generation of customized bone-like structures [30], and adipose tissue engineering [31]. Very few articles describe the application of indirect extrusion bioprinting to generate vascular structures or vascularized tissues. However, Bertassoni and colleagues applied an indirect extrusion printing approach to produce a 3D microchannel network within a photo crosslinked hydrogel [32]. They were able to generate perfusable channels within star poly (ethylene glycol-co-lactide) acrylate (SPELA), gelatin methacrylate (GelMA), poly (ethylene glycol) diacrylate (PEGDA), and poly (ethylene glycol) dimethacrylate (PEGDMA) hydrogels. Seeding these channels with cells, they demonstrated osteogenic differentiation and the formation of endothelial monolayers. 

### 3.2. Droplet-Based Bioprinting

Droplet-based Bioprinting (DBB) is a technique to deposit small droplets of biological material, including cells, growth factors, extracellular matrix components, or nucleic acids. It follows the principle of a typical office laser printer, where a small ink bubble is placed at a specific coordinate on the paper in two dimensions. The deposition of bioink bubbles on the top of another, generates the Z-axis of the 3D objects. Precise control over the printing pattern combined with its relative simplicity and the wide range of applications make DBB a very appealing printing technology. For example, DBB allows the fabrication of bifurcated tubular structures and thus enables bioprinting of vascular networks [33]. Currently, droplet sizes of 25 to 300 µm can be controlled [34]. While the DBB is not the first choice to fabricate large objects, the method can be adapted to print different cell types into one layer of a construct. Depending on the precise technology used to generate an ink bubble and deposit it, DBB can be grouped into: (i) inkjet bioprinting, (ii) acoustic-droplet ejection bioprinting, and (iii) microvalve bioprinting, with inkjet bioprinting being the most widely applied technology and with subgroups of his own [34].

#### Indirect Bioprinting Using DBB

Indirect droplet base bioprinting a blood vessel is generated by depositing endothelial cells in to create a hollow tube. Similar to indirect extrusion printing, indirect droplet-based bioprinting uses a sacrificial structure that is removed after printing is completed. In vascular bioprinting, the sacrificial structure creates the tubular lumen that can be seeded with endothelial cells.

Using an indirect microvalve DBB strategy, Lee et al. generated a perfusable model of a vascular channel. First, they printed a layer of collagen, and after its polymerization, they printed the vascular channel using a mixture of human umbilical vein endothelial cells (HUVECs) and gelatin in the ratio of 1:1. After gelation, the vascular channel was covered with another collagen layer. In a subsequent step, Lee liquified the gelatin at 37 °C and removed the material, thereby leaving a HUVEC seeded vascular channel in the collagen block. After 5 days of low-rate perfusion, the HUVEC had formed a polarized endothelial monolayer that expressed the endothelial junction protein VE-cadherin [35].

### 3.3. Light-Based Techniques

Originally designed to print electronic components, the method was adapted to print cells from the culture medium directly. Light base techniques can be divided into two separate categories two categories as digital light processing (DLP)-based and laser-based bioprinting [36].

#### Laser-Induced Forward Transfer

Laser-Induced Forward Transfer (LIFT) is a laser-assisted bioprinting (LAB) in which the bioink is placed at a specific location or substrate using a laser to generate a droplet. The technique has shown high precision and accuracy, enabling the fabrication of complex structures, using different cells and scaffold materials in various combinations. During the LIFT printing process, a stream of bionink is deposited on a metal film [37]. A pulse of energy in the range of 0.5–20 μJ generated by a nanosecond laser is then used to evaporate the bioink stream, thereby generating a droplet that is deposited on the printing bed. UV lasers with wavelengths of 193 nm, 248 nm, or near UV wavelength at 1064 nm have been implemented to create the necessary energy pulse. 

Wu et al. followed a LIFT strategy to generate branched vascular structures. With a laser pulsed at 0.5–1.5 μJ/pulse they generated bioink droplets with a diameter of 50 μm and deposited them in 50–150 μm distance from each other to form a branching vascular tree [38]. The bioink was composed of HUVEC and Matrigel^TM^. As early as one day after printing, the printed HUVEC cells had acquired a stretched phenotype, showing connected HUVEC forming a branching structure. In addition to the printed lumen, the endothelial cells formed a self-assembled secondary continuous lumen. They further found that the addition of vascular endothelial growth factor (VEGF) was essential to protect the integrity of the HUVEC and the lumen.

LIFT technique allows printing of inorganic and organic compounds with high precision in the micrometer range. Guillotin et al. combined the human umbilical vein endothelial cell line Eahy 926 and the rabbit carcinoma cell line B16 and suspended the cells in Dulbecco’s Modified Eagle Medium that was supplemented with varying amounts of glycerol, and sodium alginate or with Matrigel^TM^, or thrombin [37]. They used LIFT to print the bioink to a network with microvasculature dimensions onto a fibrinogen sheet. Using different concentrations of alginate, the authors were able to control the droplet size and thus the diameter of the printed vasculature. Alginate further functioned as a substitute to the essential extracellular matrix. An important finding was that LAB is a statistical process and that bioinks with a low cell density are unable to generate a network of continuous cells. 

### 3.4. Four-Dimensional (4D) Bioprinting

Common bioprinting is used to generate structures and arrange cells and biomolecules in the three-dimensional space. 4D bioprinting adds the factor time as the 4th dimension to the three spatial bioprinting dimensions. The term applies to bioprinted objects that, after a period of time, can change their functionality or shape as a result of cell-cell fusions or after outside stimulus [39]. For example, structures can be printed as flat objects, seeded with cells, and then rolled up or folded to a 3D shape. The stimulus to trigger the shape change can be a certain pH level, light exposure, the addition of a chemical catalyst, or a combination of similar stimuli [40]. Changing the shape of a bioprinted structure can help to overcome the limitations of regular 3D printing.

Using a 4D printing approach, Kirrilova et al. were able to generate tubes with only 20 µm diameter, which is smaller than any current conventional 3D printing technique can generate [41]. To generate a tube, they printed a layer of mouse bone marrow stromal cells suspended in a hydrogel of methacrylated alginate and hyaluronic acid flat on a glass slide. In a second step, they crosslinked the polymer with green light. Sensitive to Ca^2+^ ions, the polymer sheets changed their shape and rolled up to generate a tubular structure after being placed in solution. The possibility of generating tubular structures with small diameters renders this technique ideal to generate small vascular structures.

The group of Nakayama developed a unique strategy that allows scaffold-free 3D bioprinting [42]. Instead of a standard printing bed, where the bioink is deposited, this method uses a printing bed that consists of a paralleled array of needles, or ‘Kenzan’, with a diameter of 150 µm. A robotic arm then arranges cell-spheroids in a 3D position in the needle array. After the printing step, the spheroids will fuse, and the printed construct can be removed from the needle array [42]. The Kenzan method has been used to create scaffold-free aortas using spheroids. The printed aorta was then implanted in rat, and tissue remodeling and endothelialization were observed after five days [43]. The advantage of this method is the possibility of generating scaffold- or matrix-free tissue, which could help minimize immune rejection. On the other hand, the method is limited to printing larger constructs due to the size of the needles (150 µm) and the necessary spheroid diameter (500 µm).

## 4. Materials and Cells

Biomaterials and cells are the most important components for printing vascular and vascularized tissues. Groll et al. distinguish two different types of inks: ‘biomaterial inks’, a formulation of biomaterials and/or bioactive components (such as growth factors), and ‘bioinks’, a formulation of cells that may contain bioactive components and biomaterials [44].

When used as a supporting scaffold, biomaterials used in both inks need to exhibit specific properties. First, they should be biocompatible and resemble the material properties of native blood vessels in order to be conducive to cell viability and growth [45,46]. In addition, they should possess ideal printing properties (i.e., they should be capable of forming 3D structures with high resolution), appropriate mechanical properties (e.g., tissue elasticity, flexibility, and stability), biodegradability, biocompatibility, and low cytotoxicity [45]. Polymers possess most of those characteristics and therefore are the main biomaterial used in 3D bioprinting. However, biopolymers might harbor characteristics that one should consider when printing blood vessels (see Table 2 for a summary of the advantages and disadvantages of the main polymers). For example, a heavily crosslinked polymer will demonstrate improved printability (i.e., stiffer, higher resolution, better shape maintenance) but might exhibit reduced biocompatibility (lower cell migration, lower matrix deposition). In contrast, a less crosslinked polymer potentially might have improved biocompatibility but reduced printability [47,48].

**Figure 3:**
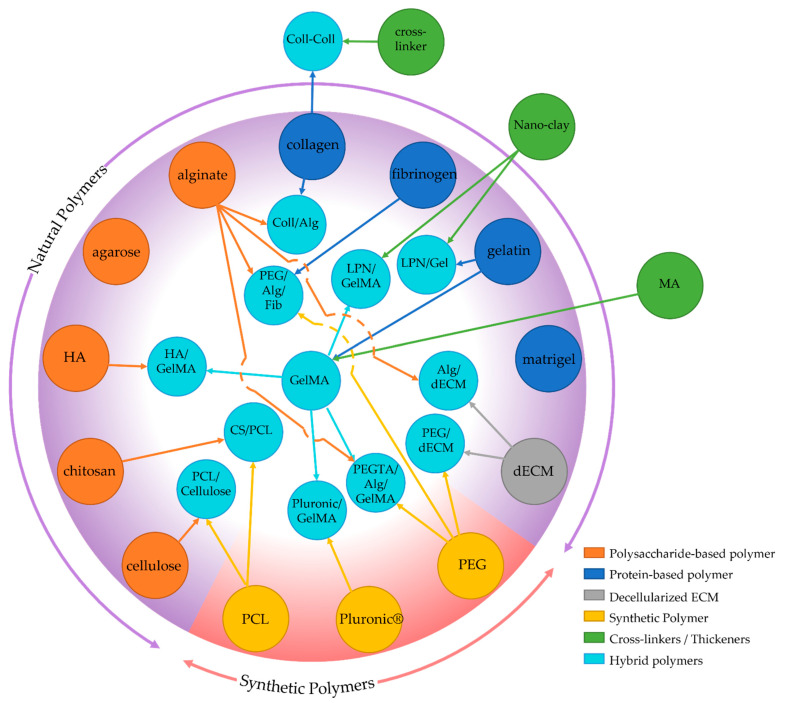
Polymers for Vascular Tissue Formation. Summary of the main natural (purple section), synthetic (red section), and hybrid polymers used. Natural polymers (polysaccharide-based [orange], protein-based [blue], and decellularized matrix [grey]) can be mixed (arrows) with natural, and/or synthetic polymers (yellow), and/or linkers or thickeners (green), to form hybrid polymers (light blue). Alg = Alginate; CS = Chitosan; Coll = Collagen; dECM = decellularized extracellular matrix; Gel = Gelatin; HA = Hyaluronic Acid; LPN = Laponite; MA = Methacrylate; PCL = poly(ε-caprolactone); PEG = Polyethylene Glycol; PEGTA = Poly(ethylene glycol) triacrylate.

Based on their origin, polymers for bioprinting inks can be divided into two categories: natural and synthetic (see Figure 3). Natural polymers are biomaterials obtained or extracted from humans, animals, fungi, and plants, while synthetic polymers are manufactured. Natural polymers possess excellent biocompatibility and can mimic the extracellular matrix (ECM), resulting in effective cell growth and function, and therefore effective tissue regeneration. However, these materials tend to encapsulate and confine cells, which limit cell-cell interaction, to have poor mechanical properties, and to show a fast degradation rate [49,50]. On the other hand, synthetic polymers, such as polycaprolactone, polylactic acid, polyethylene glycol, and polyglycolic acid, have excellent mechanical properties and tailorable processability [51,52]. In addition, control over their chemical and physical properties allows them to be customized for specific printing methods [53], making them attractive tools for tissue engineering [54,55]. However, the lack of biocompatibility, poor cell adhesion, toxic degradation products, and loss of mechanical properties during degradation limit their usability, might require chemical modifications to support cell culture and vascularization [56].

### 4.1. Natural Polymers

#### 4.1.1. Protein-Based Polymers

- Collagen

Collagen is the main component of the ECM. While there are different types of collagens, type I collagen is the most used for 3D culture due to its abundance in mammalian bodies. Collagen I provides an ideal microenvironment for EC proliferation and cell migration and has become a valuable tool in 3D bioprinting of vascularized tissue, in concentrations ranging from 1.2 to 1.9 mg/mL [57].

Collagen is non-immunogenic and offers rapid gelation under specific conditions, making it an ideal candidate for extrusion bioprinting. However, it suffers from a lack of good mechanical properties and low cell adhesion when unmodified. To improve the printability of collagen gels, biocompatible crosslinkers can be added to the biopolymer [58]. For example, Kim & Kim have crosslinked bioinks composed of collagen and Caco-2 cells or human umbilical vein endothelial cells (HUVECs) to form vascularized intestinal villi. Both cell types proliferated in their region, and cell-cell interaction could also be demonstrated [59]. Pataky et al. have incorporated alginate in collagen gels to speed up the gelation and improve the fidelity of the printed structures [60]. Fast gelation of the alginate offers mechanical support for the collagen component. After gelation of the collagen, the supportive alginate could be removed, while collagen kept the defined shape. Although one can see the advantage of this technique for blood vessel fabrication, to our knowledge, nothing has been done in this direction.

- Gelatin

Gelatin is a fibrous protein derived from irreversibly hydrolyzed collagen. Gelatin possesses various advantages that make it very appealing for scaffold fabrication. It has high water absorption, excellent biocompatibility, excellent biodegradability, and is non-immunogenic. It is often used as a sacrificial layer due to its modifiable thermoresponsive properties. However, gelatin-based inks have a low printing resolution (>100 µm) and shape fidelity, limiting its use for 3D bioprinting. Thickeners, such as nanoclays [61] and transglutaminase [62], have been used to improve gelatin printing properties. However, although thickeners can improve the fidelity of the printed structure, they do not improve the resolution of the hydrogel. The resolution can be improved by the addition of crosslinkers, such as methacrylate (MA), which helps to form a gel with higher mechanical stability. Gelatine MA (GelMA) can be processed more easily and demonstrates good cell viability, proliferation, and spreading [63,64,65]. However, the presence of toxic photoinitiators and UV exposure during the gel formation process can affect cell viability, which is a significant disadvantage to the use of MA. Recently, Cidonio et al. have incorporated synthetic nanoclay laponite (LPN) into GelMA and loaded the gel with VEGF. In vascular chick embryos, VEGF-loaded LPN-GelMA constructs demonstrated higher blood vessel penetration than VEGF-loaded GelMA scaffold [66].

- Fibrinogen

Fibrin has been used in vascularization scaffold fabrication because of its inherent cell-adhesion capabilities. It promotes EC proliferation by directing cells towards growth factors, such as VEGF and FGF. It also can be used as a scaffold to support EC growth. When used as a single component, fibrin exhibits poor mechanical properties and can degrade rapidly. Therefore, the protein has been blended or attached to other biomaterials, such as alginate and PEG, which have better printing characteristics. Maiullari et al. (2018) fabricated a vascularized cardiac tissue using a bioink composed of alginate, PEG-fibrin, HUVECs, and iPS-derived cardiomyocytes [67]. More recently, Piard et al. (2019) have used gelatin as a support material to compensate for fibrin mechanical degradation properties [68]. Using pressure-based extrusion 3D bioprinting, they fabricated an osteo-like scaffold with two separate cell populations (osteogenic and vasculogenic) encased in fibrin bioink. This construct induced a significant expression of angiogenic factors in vitro and increased blood vessel formation in vivo, 14 days after subcutaneously implantation in rats.

- Matrigel™

Matrigel™ is a mixture of ECM proteins isolated from Englebreth-Holm-Swarm mouse sarcoma cells [69,70]. It is used to mimic ECM of various cancer and stem cell lines and helps to maintain the stem cells in an undifferentiated state [69]. Extrusion-based printing has been used to form lumen in primary rat hepatocyte-loaded Matrigel™ gel. HUVEC have then been seeded in the microlumens by injection and then perfused. Matrigel™ has been shown to promote tissue vascularization. Compared to culture without HUVEC, vascularized engineered tissue helped to improve hepatocyte metabolic function, albumin secretion, and urea synthesis [71]. Laser-guided direct-write (LGDW) technique has been used to print a HUVEC 3D vascular network on Matrigel™, with ECs forming elongated tube-like structures in vitro [72]. However, the long fabrication process, potential laser-induced cell damages, and low scalability limit the application of Matrigel™ for vascularized tissue fabrication. Recently, Matrigel™ has been used to fabricate vascularized cardiac patches. The patches were culture in vitro and prevascularized in the omentum before their implantation in the heart. After four weeks, the implanted patches showed improved structural and electrical integration [73].

#### 4.1.2. Polysaccharide-Based Polymers

- Cellulose

Cellulose is the most abundant organic polymer on earth [74]. It is a structural component of the primary cell wall of green plants, oomycetes, many forms of algae, and in the biofilm secreted by some species of bacteria [75]. Because of its natural origin, cellulose is environmentally friendly and is considered the safest material on earth [76]. It is a polymer consisting of a linear chain of 100 to 1000 β(1→4) linked D-glucose units. Cellulose possesses good mechanical strength, biocompatibility, and biodegradability. However, it is a hydrophilic material with high hydrogen bonds and Van der Waals forces that make the dissolution difficult.

One way to overcome this difficulty is to use active cellulose-secreting bacteria to fabricate cellulose hydrogel. For example, Shin et al. (2019) described the fabrication of complex 3D structures with a bioink that contained active bacteria (*Gluconacetobacter xylinus, G xylinus*) and cellulose nanofiber [77]. It allowed control over the topology and the dimensional stability using a solid matrix-assisted 3D printing process (SMAP) and polytetrafluoroethylene (PTFE) microparticles as printing matrices. The bacteria G. xylinus produced a cellulose hydrogel at the medium’s surface after the bacterial metabolism was activated by oxygen that was perfused through the PTFE matrix. The fibroblasts’ culture on the lumen’s apical surface of perfusable tube-like structures suggests possible applications in vascular tissue engineering and the fabrication of artificial blood vessels.

Cellulose can also be mixed with other chemicals to adjust its properties. For example, when combined with poly(3-caprolactone) (PCL), cellulose acetate (CA) presents better mechanical properties while maintaining suitable biocompatibility and biodegradability, support of cell adhesion, proliferation, and migration. In addition, when aligned, PCL/CA nanofibers can guide and regulate the organization of HUVECs, and in vitro, facilitate capillary-like tube formation and scaffold prevascularization. After implantation, blood vessels from the host penetrated the prevascularized scaffold and integrated with the vascular network [78].

- Alginate

Alginate is a polysaccharide isolated from brown algae. Alginate polymerizes rapidly and, in the presence of divalent ions (e.g., Ca^2+^, Mg^2+^, and Ba^2+^), can form hydrogels. Hydrophilic chains with a large holding capacity for water help to form a polymerized hydrogel. Its porous internal structure permits the diffusion of soluble molecules, making it a material of choice for tissue engineering. Alginate’s short polymerization time allows the printing of shapes using extrusion-based and inkjet bioprinting [65,79]. In addition, alginate can be modified to control its degradability. The shear-thinning properties of alginate help to minimize the shear stress effect on suspended cells during printing, thereby increasing cell viability.

Despite various advantages, ECs cultured in alginate hydrogels displayed low cell viability and failed to form vascular networks. To enhance cell survival and vascularization, alginate has been mixed with biomaterials exhibiting greater biocompatibility, such as collagen and fibrin with or without VEGF [80]. Alginate’s low cell attachment properties are associated with reduced low vascular network formation tendency. Incorporating RGD peptides into the alginate hydrogel can drastically improve cell attachment [81].

In addition to being a scaffold for vascular network formation, alginate has been used as a sacrificial layer to form perfusable tubular structures with high shape fidelity [82]. After tube formation, alginate can be removed by chemically releasing the divalent ion crosslinker, either through an increase of monovalent cation concentration [83] or by adding a chelator [84] to the surrounding media.

- Hyaluronic acid (HA)

Hyaluronic acid (HA) is a polymer of disaccharides composed of (1–4)-linked β-D-glucuronic acid and (1–3)-linked β-N-Acetyl-D-glucosamine. It is a key component of the ECM that supports cell growth and viability. Unmodified HA has a high viscosity and low formability, mechanical properties that limit its use in bioprinting. The mechanical properties of HA can be improved by adding GelMA [72]. Adding methacrylate groups can make HA photopolymerizable, while retaining the biological activity of HA to promote endothelial cell proliferation [85]. Zhu et al. demonstrated the printing of a prevascularized tissue construct that was able to support the formation of an endothelial network after implantation [86].

- Agarose

Agarose is a linear polymer consisting of the repetition of agarobiose units, a disaccharide of D-galactose and 3,6-anhydro-L-galactopyranose, derived from red seaweed [87,88]. Agarose can create a thermo-reversible hydrogel with gelling and melting temperatures ranging between 34°–52° and 85°–95°, respectively, depending on the type of agarose. High melting temperature and low cell adhesion properties limit the use of agarose as bioink. To promote cell adhesion and proliferation, agarose must undergo chemical modification or combine with other polymers, such as gelatin [89]. Therefore, agarose has been used as a molding template for the fabrication of small-diameter vascular constructs. In this model, Norotte et al. (2009) printed multicellular spheroids layer-by-layer, concomitantly agarose rod-molding templates [90].

- Chitosan

Chitosan is a linear polysaccharide of β-(1→4)-linked D-glucosamine and N-acetyl-D-glucosamine that are randomly distributed. It is prepared by treating shrimp and other crustaceans’ chitin shells with an alkaline chemical, such as sodium hydroxide. Chitosan hydrogels offer strong biocompatibility, biodegradability, have low toxicity and non-immunogenicity, and can be easily modified [91]. Furthermore, chitosan gels are cationic, a unique property that allows mixed biopolymers formation through interactions with ECM structural molecules, glycosaminoglycans, and glycoproteins [91,92], or anionic biopolymers, optimizing the mechanical or biological properties [93]. However, due to the poor mechanical properties of chitosan, it has to be mixed with other materials. For example, Du et al. developed a vertically graded chitosan/poly ɛ-caprolactone (CS/PCL) nanofibrous vessel scaffolds fabricated with chitosan and PCL by sequential quantity grading co-electrospinning, loaded with heparin to immobilize vascular endothelial growth factor (VEGF) in the gradient CS/PCL [94]. The number of heparinized chitosan nanofibers increased gradually from the tunica adventitia to the lumen surfaces in the gradient CS/PCL wall of tissue-engineered vessels. The vertical gradient heparinized CS/PCL nanofibrous scaffold was then seeded with HUVEC on the luminal side and smooth muscle cells on the other side. Interestingly, the construct exhibited antithrombic activity, promoted cell proliferation and accelerated re-endothelialization. This suggested that the scaffold could provide an approach to create small-diameter blood vessel grafts.

#### 4.1.3. Decellularized ECM-Based

Decellularized ECM (dECM) is the ECM scaffold that remains after cells are removed from a tissue. It contains biological, structural, functional, and topographical cues required to promote vascular tissue growth [26,95]. Different tissues have their specific ECM composition. Therefore, dECM of targeted tissue supports cellular growth and functions better than other biomaterials [96]. It further promotes native vessel-like structures after transplantation [97]. More interestingly, blood vessel constructs prepared with dECM prevent thrombosis and intima hyperplasia for up to 70 [98] or 30 days [99] after implantation, respectively.

Despite these advantages, dECM’s low mechanical properties and slow gelation process limit its use. The mechanical properties can be improved by integrating crosslinkers such as PEG-based crosslinkers or methacrylate [100,101]. In addition, slow gelation behavior can be modified by incorporating fast-gelling materials, such as alginate. Alginate/dECM presents favorable properties for vascularized tissue formation; dECM supports angiogenesis and the vascularization of the scaffold [102], while alginate’s rapid gelation process allows 3D printing of blood vessels [96].

Due to the nature of dECM, it is necessary to consider the potential for acute rejection induced by non-autologous ECM, as well as accidental pathogen transmissions. Patient-specific dECM might therefore be more convenient as a scaffold material than other biomaterials that are more readily available [26,95].

### 4.2. Synthetic Polymers

A variety of synthetic polymers have been used for 3D bioprinting, such as polylactic acid (PLA) [52,103,104], polycaprolactone (PCL) [105], polyethylene glycol (PEG) [84,106], polypropylene fumarate (PPF) [107], and Pluronic^®^ F127 [108]. Among these polymers, PEG and its derivatives are probably the most explored for soft tissue engineering [109]. Pluronic^®^ F127 is one of the most promising polymers as sacrificial templates in vascular tissue engineering.

- Polyethylene Glycol (PEG)

PEG, also known as poly(oxyethylene) or poly (ethylene oxide) (PEO), is a hydrophilic, biocompatible, non-immunogenic synthetic polymer composed of a linear and a branched structure. PEG hydrogels are naturally nonbiodegradable, and they can be modified to enhance degradation by incorporation of degradable segments such as PLA [110] or PCL [111]. The lack of cell-adhesive domains within PEG inhibits cell-attachment and proliferation and requires modification of PEG with other biomolecules to accommodate cell culture and tissue growth.

PEG is often modified by incorporation of acrylate groups to create a photopolymerizable PEG di-acrylate (PEGDA) or PEG-tetra-acrylate (PEGTA), in which cells can easily be encapsulated, using UV/visible light assisted printing techniques, with polymerization times between 20 and 60 s [112]. PEGDA and PEGTA are frequently used in combination with more cell-friendly biomaterials to enhance their biocompatibility. For example, Jia et al. designed a cell-responsive ink consisting of GelMA, alginate, and 4-arm PEGTA combined with a multilayered coaxial extrusion system to achieve direct 3D bioprinting of perfusable vascular constructs [84]. This bioink combined the benefits of both natural and synthetic biomaterials, displaying favorable characteristics for the culture and proliferation of encapsulated vascular cells while maintaining mechanical properties suitable for printing complex perfusable vascular constructs.

- Pluronic^®^

Pluronic^®^ F127 is a polymer with demonstrated potential for vascular tissue bioprinting. It is a tri-block copolymer consisting of a hydrophobic poly (propylene oxide) (PPO) segment and two hydrophilic PEO segments arranged in a PEO-PPO-PEO pattern. This new high molecular non-ionic surfactant class is a synthetic thermoplastic polymer with a gelation temperature related to its concentration and structure. Pluronic^®^ F127 offers a high resolution of the printed construct, but it possesses weak mechanical properties, quick degradation rates, and rapid dissolution in aqueous solutions. The polymer is associated with poor cell viability, significantly limiting its application as a scaffold in bioartificial organ 3D printing. UV/light-based cross linkability can be created by acrylation or methacrylation of the Pluronic^®^. By combining thermal reversibility of gelation and photocrosslinkability within Pluronic^®^ F127-based hydrogels, Wu et al. developed a 3D perfusable microvascular network [55]. Unmodified Pluronic^®^ is commonly used as fugitive ink, as it can be easily printed and removed under mild conditions. For example, Kolesky et al. (2014) used Pluronic^®^ F127 to print vascular channels [113]. After gelation, GelMA was used as a bulk matrix and cell carrier. After photopolymerization of GelMA, the fugitive ink of Pluronic^®^ F127 was removed, yielding open microchannels for the fabrication of HUVEC-based vascular embedded constructs.

**Table 2:** Advantages and Disadvantages of the Main Polymers.

Polymer	Advantages	Disadvantages	References
Collagen	non-immunogenic rapid gelation	lack of good mechanical properties low cell adhesion	[56]
Fibrinogen	inherent cell-adhesion capabilities		[67]
Gelatin	high water absorption excellent biocompatibility excellent biodegradability non-immunogenic modifiable thermoresponsive properties	low printing resolution (>100 µm) shape fidelity	[61,62]
Matrigel	Promote vascularization		[71]
Alginate	diffusion of soluble molecules short polymerization time shear-thinning properties	low cell viability low cell attachment	[65,80]
Agarose		high melting temperature low cell adhesion low proliferation properties	[89]
Hyaluronic Acid	cell growth Viability	high viscosity low formability	[85]
Chitosan	biocompatibility biodegradability low toxicity non-immunogenicity easily modifiable	poor mechanical properties	[91]
Cellulose	biodegradability good mechanical strength biocompatibility	difficult dissolution	[76,79]
dECM	cellular growth better than other biomaterials functions better than other biomaterials promotes native vessel-like structures prevent thrombosis and intima hyperplasia	low mechanical properties slow gelation process	[26,96,101,102]
Pluronic	high resolution of the printed construct	weak mechanical properties quick degradation rates rapid dissolution in aqueous solutions poor cell viability	[55,108,113]
PEG	hydrophilic biocompatible non-immunogenic	naturally nonbiodegradable inhibits cell attachment Inhibit cell proliferation	[110,111]

### 4.3. Cell Sources

Cell choice is essential to print functional tissues (see Table 3). To generate vascular and vascularized tissues by bioprinting, parenchymal cells and non-parenchymal cells (i.e., cells with structural, supportive, or barrier functions) have to be incorporated. These cells must maintain cellular homeostasis and their potential for self-renewal while providing their designated functions (parenchymal or not) after printing to recapitulate the entire tissue physiology. Moreover, cells must be robust enough to support the printing process while having control over the cells to prevent excessive proliferation hyperplasia or apoptosis. For translational applications, autologous cells are preferable to prevent graft rejection. Therefore, patient-specific cells, including tissue-specific primary cells, mesenchymal stem cells (MSC), and induced pluripotent stem cells (iPSC), are considered potential sources for tissue printing.

Many adult primary cells isolated from patients show limited proliferation capacities and functions, limiting their use in tissue engineering approaches. MSC offer an alternative to primary cells. Mainly isolated from bone marrow, peripheral blood, adipose tissue, placenta, and umbilical cord, they exhibit multipotency and can be differentiated into osteogenic, chondrogenic, tenogenic, myogenic, adipogenic, and stromal lineages [114,115,116]. Using established protocols, MSC have the potential to be safe and a promising resource for clinical applications. iPSCs are genetically reprogramming from differentiated adult cells, such as dermal fibroblasts and peripheral blood [117,118]. They are capable of indefinite self-renewal and show pluripotent characteristics. Many cell types have been successfully differentiated from iPSC, including cardiomyocytes, chondrocytes, osteoblasts, hepatocytes, neural cells, and endothelial cells [119]. While the potential of iPSC has been demonstrated in proof-of-concept studies, advanced cell differentiation protocols need to be developed to culture and enrich specific cell types. Functionally differentiation of patients’ iPSC is a necessary prerequisite for personalized tissue bioprinting and other regenerative medicine applications.

**Table 3:** Advantages and Disadvantages of the Main Cell Sources.

Cells	Source	Advantages	Disadvantages	References
Tissue-specific primary cells	Tissue-specific stem/progenitor cells (e.g.; brain, spinal cord, heart, skin, hair follicle)	Reduced risk of rejection (autologous)	Small quantities especially medically fragile patients limited proliferation capacity difficult to obtain	[120,121]
Mesenchymal stem cells (MSC)	Bone marrow Peripheral blood Adipose tissue Placenta Umbilical cord	Multipotency Immunomodulatory properties, Well-characterized	Differentiation potential decreases with increasing age	[114,115,116,120]
Induced pluripotent stem cells (iPSC)	Reprogram from dermal fibroblasts or peripheral blood	Pluripotentcy, indefinite self-renewal Patient-specific	Risk of in vivo teratoma formation Streamlined differentiation protocols needed Possible genetic mutations	[117,118,122]

## 5. Tissue Examples

**Figure 4:**
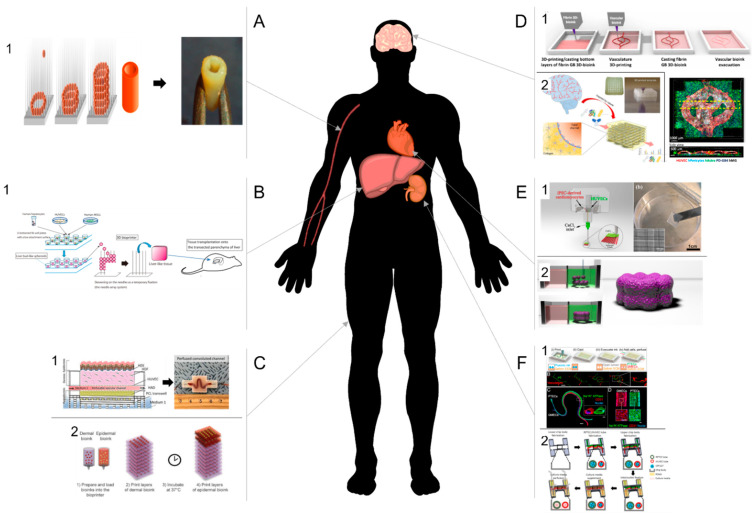
3D bioprinted scaffold for tissue vascularization (**A**) or vascularized tissues (**B**–**F**). (**A**) Cardiovascular system. (1) A tubular tissue was printed from multicellular spheroids (MCS) using needle array bioprinting system (left). After fusion of the spheroids, the tubular structure can be removed from the needle array and used as a blood vessel [43]. (**B**) Liver. (1) A liver-like tissue was constructed from liver bud-like spheroids composed of hepatocytes, mesenchymal stem cells, and endothelial cells, on a needle array. After fusion of the spheroids, the tissue was removed and implanted onto the transected parenchyma of mouse liver [134]. (**C**) Skin. (1) Schematic representation (left) of a bioprinted perfusable vascularized human skin tissue composed of a hypodermal pre-adipocyte compartment containing the vascular channel and covered with a dermal layer and topped with printed primary human epidermal keratinocytes. Pictures of the perfused tissue (right) [144]. (2) Schematic representation of a bioprinted human skin equivalent composed of multiple dermal layers (dermal bioink) and two epidermal layers (keratinocytes bioink) [143]. (**D**) Brain. (1) Schematic representation of the printing process for the fabrication of an artificial blood-brain barrier (BBB-top). After casting a bottom layer of glioblastoma(GB)-fibrin bioink, a perfusable blood vessel was bioprinted with a sacrificial ink. The vasculature network was then covered with GB-fibrin bioink. After removal of the sacrificial ink, the vasculature system was perfused. Picture (bottom right) of the confocal imaging of the bioprinted BBB [140]. (2) Schematic representation and picture of bioprinted BBB. The vascularized neural construct was fabricated from a bioprinted interconnected blood vessels wrapped with primary pericytes, astrocytes [137]. (**E**) Heart. (1) Schematic representation of the bioprinting process of a heart patch composed of one layer of iPSC-derived cardiomyocytes and one layer of HUVECs (left). Picture of the patch after printing (right) [67]. (2) High cell-density cardiac fibrosis model printed from spheroids within self-healing hydrogels [125]. (**F**) Kidney. 3D bioprinted proximal tubule (PT). (1) Top—Schematic representation printing process of a vascularized proximal tubule (PT). Both PT and vascular channels are printed with a Pluronic-based sacrificial ink and covered with a gleatin-fibrinogen gel. After removal of the sacrificial ink, cells can be added and cultured in the lumen (top). Picture of the fabrication process; Middle—Fluorescence images of the PT (green) and the vascular (red) channel; Bottom—confocal microscopy images of both channels [148]. (3) PT and blood vessel mixed with decellularized kidney extracellular matrix (KdECM) were printed in coaxial channels to build a 3D vascularized renal proximal tubule-on-a-chip [149].

Vascularization is essential to preserve tissue functions. A robust perfusable vascular network is essential to enable gas exchange, nutrient diffusion, and removal metabolic products in bioprinted tissues. To maintain cell physiological viability in vivo, the distance between a cell population and a blood vessel cannot exceed 100–200 μm [123]. For tissue survival it is therefore essential to precisely incorporate the vasculature or to create a perfusable system within 3D printed tissue. Currently, two main approaches have been established to create vascularized 3D printed tissue. One option is to induce angiogenesis in 3D printed tissue, the other to directly print a vascular channel. In the section below, we will present current attempts to develop vascularized 3D printed tissues (see Figure 4 and Table 4). 

### 5.1. Cardiovascular Tissues

The cardiovascular system, or circulatory system, includes heart and other organs, blood vessels, and the lymphatic system. As described throughout this review, blood vessels are essential for the viability and functionality of native and engineered tissues and organs. Here we will focus on the printing of large blood vessels (150 µm and larger), while other sections will focus on the integration of capillaries into printed tissues. Various methods have been developed to fabricate large blood vessels. One of the earliest attempts to use a commercially available printer to generate short smooth muscle cell (SMC) tubes was made in 2005 by Kesari et al. [124]. They 3D printed alginate tube constructs of with 2 mm diameter and 2 mm length using a modified thermal inkjet printer. The printed tubes exhibited vasoreactivity to agonists while maintaining mechanical strength; demonstrating the potential of this drop-based printing method to construct functional tissue-engineered vessels. Droplet-based bioprinting however, is a slow process with limitations on print resolution, materials, and cells. To overcome said limitations, extrusion-based methods were developed as an alternative. For example, Miller et al. introduced a sacrificial molding method to generate a vascular network with interconnected vessels of different diameters ranging from 750 to 150 µm [71]. The biocompatible sacrificial material made from mixed carbohydrates was 3D printed into a rigid filament network. The material is compatible with various natural and synthetic polymers, including PEGDA, PEG-RDS, fibrin, Matrigel^TM^, and agarose gels. After polymerization, the sacrificial layer was dissolved, the cylindrical network seeded with endothelial cells and perfused with blood under high-pressure pulsatile flow.

More recently, Itoh et al. developed a scaffold-free printing method to fabricate tubular structures with 1.5 mm diameter lumen. After in vitro culture, the tubules were implanted into the abdominal aortas of nude rats. After five days, the grafts showed successful incorporation into the aortas and had undergone tissue remodeling. Tubular lumens were found to be enlarged, tubular walls thinned, while apical lumen maintained a layer of host endothelial cells [43].

In addition to focusing on the fabrication of perfusable tubular constructs, notable efforts have been made towards the fabrication of vascularized cardiac tissue. Engineering of cardiac tissue was seen as a potential therapeutic strategy for to treat myocardial infarction and heart failure. In 2018, Maiullari et al. bioprinted a heterogeneous, multicellular heart tissue patch by including HUVECs and iPSC derived cardiomyocytes (iPSC-CM) [67]. Cells were encapsulated in alginate and PEG-Fibrinogen (PF) hydrogel, cell populations were extruded alternating to form heterogeneous structures with different spatial organizations. After in-vivo subcutaneous implantation, Maiullari found that the host’s vasculature invaded and integrated into the bioprinted constructs. Although iPSC-CM could not form any functional organization in vitro, the invading vessels formed branched vascular capillaries, and they observed a better organization and orientation of the CM [67]. These results suggested that bioprinted constructs have the potential to mature into vascularized functional tissues in vivo.

Later, cardiac spheroids generated by iPSC-CMs and primary human cardiac fibroblasts (CFs) served as cell sources for cardiac tissue bioprinting in several studies. Daly et al. bioprinted cardiac spheroids within self-healing gel into a ring pattern to form a microtissue by spheroid fusion [125]. They further mixed iPSC-CMs and CFs at 4:1 and 1:4 ratios to denote healthy tissue and the focal cardiac fibrosis disease model respectively. The slower depolarization as well as reduced sarcomere formation, calcium flux and contraction amplitude were observed in scarred spheroids [125].

Cardiac spheroids generated from iPSC-CM and primary human cardiac fibroblasts (CF) served as a cell source for cardiac tissue bioprinting in several studies. For example, cardiac spheroids were used to fabricate cardiac tissue by sacrificial writing into functional tissue (SWIFT) technique (an extrusion-based bioprinting technique) [126]. In this study, the beating cardiac spheroids generated from patient-specific iPSC-CM and primary ventricular normal human cardiac fibroblasts were mixed with human neonatal dermal fibroblasts and rat tail collagen I to generate a cardiac tissue matrix. A perfusable branching channel architecture was then printed within the cardiac tissue matrix [126]. They observed a 20-fold contractility increase after starting perfusion and that spontaneous contraction was responsive to calcium or drug treatment. The authors also printed an arterial vascular network according to patient-specific cardiac structural data within a cardiac tissue matrix, demonstrating a potential utility for therapeutic applications [126].

Lee et al. used collagen for bioprinting structures of human hearts at various scales from capillaries to whole organs [127]. In this study, they used a extruded 3D-bioprinting approach to precisely generate collagen filaments and accurately reproduce complex organ structures by freeform reversible embedding of suspended hydrogels (FRESH) consisted of gelatin microparticles. In a first step, a small coronary artery-scale linear tube was printed from FRESH and connected with a pulsatile perfusion system. Next, a collagen gel loaded with mouse skeletal muscle cell line C2C12 was cast around the printed coronary artery tube and tubes subsequently perfused. A space-filling branching network was created to generate a multiscale vasculature. As a result, vessels from the left coronary arteries extended into a dense arterial network with smaller diameters [127]. Next, they FRESH-printed a model of the heart’s left ventricle in an ellipsoidal shell with a central section of cardiac cells, using human embryonic stem cell-derived cardiomyocytes (hESC-CM) and cardiac fibroblasts as well as internal and external collagen shells. The authors observed contracting hESC-CM throughout the entire printed structure and anisotropic calcium wave propagation under the side point stimulation [127]. Lee et al. also printed an adult human sized tri-leaflet heart valve, connected it to a peristaltic pump, and observed cyclical opening and closing of the valve leaflets, suggesting its mechanical function [127]. Lastly, they printed a neonatal scaled human heart, including atrial and ventricular chambers, trabeculae, and pulmonary and aortic valves, proving the feasibility to recapitulate the structural, mechanical, and biological properties of the native tissue [127].

### 5.2. Liver

The liver is a highly vascularized organ with a role in detoxification, protein synthesis, and carbohydrate/lipid/drug metabolism. The hepatocytes, which account for 80% of the total liver volume, line up to form hepatic plates, separated by a fenestrated, discontinuous endothelial system called sinusoids. As blood flows inward from the hepatic artery and the portal vein to the hepatic vein, a sequential exchange of compounds and tissue morphogens results in a graded microenvironment along the vasculature [128]. The flow of blood along the portal-central axis establishes concentration gradients of oxygen and hormones in the hepatic plates, which causes different enzyme content in the hepatocytes of the periportal zone from those in the perivenous zone. Therefore, specialized hepatocytes in different zones serve as various compounds’ metabolism as well as drugs metabolism by cytochrome P-450 in the liver [129,130]. 

In a proof-of-concept study, Bhise et al. demonstrated the utility of a liver-on-a-chip platform for drug toxicity screening [131]. In their study, they encapsulated preformed human HepG2/C3A spheroids in gelatin methacryloyl (GelMA) hydrogel, and bioprinted the spheroids in multilayers using a dot array on a PDMS chamber. The liver spheroids bioreactor was then perfused with media continuously via syringe pump. The constructs could be long-term cultured up to 30 days. When treated with 15 mM acetaminophen, the cellular metabolic activity and hepatic biomarkers secretion, including albumin, A1AT, transferrin and ceruloplasmin were significantly decreased within 6 days. Which is similar to data reported on animal or in vitro models [131].

Nguyen et al. used primary human hepatocytes, hepatic stellate cells, and HUVEC cells to construct a scaffold-free, 3D-bioprinted vascularized liver tissue for drug screening [132]. The parenchymal hepatocytes filled each compartment, while the non-parenchymal cells, stellate cells and HUVEC, were extruded on the border region. Over time, extensive vascular networks of activated stellate cells that expressed desmin and α-SMA could be observed at the tissue periphery. Physiological levels of Adenosine triphosphate (ATP), albumin and cytochrome P450 were also sustained during the four weeks of in-vitro culture [132]. The vascularized hepatic tissues were then used to assess the toxicity of trovafloxacin and to determine the toxicity of clinically relevant doses, which could not be evaluated with conventional hepatic cultures. This powerfully demonstrated the importance of vascularization in 3D-printed liver tissues for the development of personalized drug screening [132].

In addition to be a platform for drug screening, 3D printed liver tissue was also tested in an in-vitro disease model for liver fibrosis. To investigate the role of Kupffer cells in the initial response of liver fibrogenesis, Norona et al. fabricated 3D printed liver tissues comprising primary human hepatocytes, human stellate cells, human umbilical vein endothelial cells with or without the addition of primary human Kupffer cells [133]. Markers for liver injury, hepatic function, as well as collagen deposition, inflammatory cytokines secretion and gene expression profiles were evaluated to delineate the impact of Kupffer cells in TGF-β1- and methotrexate-induced fibrotic injury. The findings delivered valuable information for the development of an in vitro model to identify the compounds for liver fibrosis therapeutic assessment [133].

Recently, several studies reported the transplantation of bioprinted liver constructs in animals with experimental liver injury, with animals showing normal post-transplant liver function [134,135]. For example, Yanagi et al. printed scaffold-free 3D liver tissue from liver bud-like spheroids (LBS) in combination with HUVEC, hMSC, and human hepatocytes (hHep) [134]. The assembled LBS were bioprinted on the needles of a pinholder using a needle array system. After LBS fusing, the needles were removed, and the arranged structure self-organized into liver-like tissue. The authors then transplanted the biofabricated hepatic tissue onto the transected liver parenchyma of nude rats’. Seven days after transplantation, the expression of albumin and CYP3A4 was detected, further vascular in the graft was observed. In addition, although the fibrosis expanded past 28 days after surgery, CK 19 positive cells were observed, indicating the potential formation of bile ducts. The results suggested that the blood supply to the grafts was insufficient, and that pre-vascularization might be necessary for thicker tissue layers [134]. To improve transplant anastomosis and to reduce ischemia time, Stevens et al. created a pre-vascularized hepatic tissue. The bioprinted liver tissue was fabricated from primary human hepatocytes, HUVEC, and normal human dermal fibroblasts (NHDF) in a degradable hydrogel [135]. To create the pre-vascularization in the hepatic tissue, a bioink composed of endothelial cords suspended in collagen hydrogel was printed, then primary human hepatocyte-NHDF aggregates were suspended in fibrin and added over the printed endothelial cords. After polymerization, the tissue was transplanted onto the mesenteric fat of *Fah−/−* NOD *Rag1−/− Il2rgnull* (FNRG) mice. Interestingly, vascularization of the transplant tissue was observed in mice with experimental liver injury, but not in controls. Following vascularization, the graft significantly developed, forming bile-duct-like structures and secreting functional hepatocyte specific proteins including albumin, transferrin, A1AT, and fibronectin [135]. More recently, Grigoryan et al. further established intravascular hepatic hydrogel carriers with perfusable vasculature to implant in FNRG mice [136]. The hydrogel carrier was printed by PEGDA and GelMA mixture, then a HUVEC-collagen mixture was seeded on the open channels to form the endothelial cell cord. Rat primary hepatocytes and NHDF hepatic aggregates were suspended in fibrin and cast over endothelial cell channels to fabricate a hepatic hydrogel carrier with perfusable vasculature. The albumin promoter activity was sustained 14 days post implantation in FNRG mice, and host blood cells infiltration was detected in the graft, demonstrating the potential utility of the printed hepatic hydrogel carriers in clinical therapy [136].

### 5.3. Brain

The human brain metabolizes roughly 25% of the oxygen and 60% of the glucose in the body to maintain its functionality in a resting state. Adequate blood supply is therefore critical for the development of 3D brain tissue in vitro. One of the most critical structures in the brain vasculature is the blood-brain barrier (BBB), a unique and selective physiological barrier that controls transport between the blood and the neural tissue. Yue et al. developed 3D vascularized neural constructs to reconstitute BBB function ex-vivo [137]. A PCL/PLGA tubule network was printed with brain endothelial cells and perfused to generate 3D interconnected blood vessels. The vasculature network was wrapped with primary pericytes, astrocytes, and neurons within a collagen matrix to form a vascularized neural construct [137]. To study BBB function and drug permeability, peptides Angio2, TAT, and HoxA-13 and biological molecules NGF and Huperzine A (with or without BBB penetrating capability) were circulated through the printed vasculature network and their BBB penetration levels were measured. BBB penetration characteristics of the tested molecules in the printed neural construct were similar to their in vivo behavior in the human brain. The vascularized neural constructs also showed significantly improved neuronal differentiation after perfusion with HuperzineA, an experimental drug for the treatment of neurological disorders [138]. This work demonstrated the reconstitution of BBB function in vitro and offered a novel method to examine the BBB-penetrating potential of drug candidates [137].

Glioblastoma (GB) is the most common primary malignant brain cancer with a low 5-year survival rate and poor prognosis. The aberrant microvasculature hyperplasia composed of endothelial cells, smooth muscle cells, and pericytes observed in GB microenvironment is the primary factor that causes chemo-resistance [139]. Using a 3D bioprinting strategy to build an ex vivo vascular GB model is important to study GB tumor biology. Recent advances in bioprinting allowed for fabrication of in-vitro brain tumor models for the development of personalized treatment. For example, Neufeld et al. created a perfusable GB model for in-vivo mimicry of the tumor microenvironment [140]. The GB model was printed in several steps. First, a bottom layer composed of patient-derived GB cells was mixed with human astrocytes and microglia and suspended in fibrin bioink was printed. Then the vascular network was printed using direct extrusion printing. Finally, a layer of GB bioink was applied to cover the vascular network. A mixture of HUVECs and primary human microvascular brain pericytes (hPericytes) was injected into the vascular channel to build a perfusable blood vessel, mimicking the BBB [140]. The printed system was capable of transporting nutrients and catabolites through the vasculature to the brain structures. Two GB subclones from a human GB cell line were used. When cultured in vitro, both clones showed similar cell proliferation rate and drug sensitivity, but when injected in a mouse, one clone exhibited dormant and the other fast-growing characteristic. 

When used in the 3D printed model, the fast-growing GB also showed enhanced cell proliferation, invasion, and a reduced inhibition efficacy of P-selectin inhibitor, similar to the behavior in the murine model. RNA-seq analysis of transcriptional profile of the 3D-bioprinted GB model and in-vivo GB, confirmed the similarity between the mouse model and the characteristics of the printed 3D tumor model [140]. Accordingly, these results suggest that the perfusable 3D-printed tumor model has the potential to reduce or replace certain animal models and could serve as a platform to develop personalized drug screening [140].

### 5.4. Skin

The skin has a barrier function and protects the body from dehydration and microorganism infection. Chronic, non-healing wounds caused by burns, trauma, or diseases are clinically relevant because they pose a huge burden to the patient and the healthcare system. The current treatment for chronic wounds includes biocompatible wound dressings, engineered skin grafts, and split-thickness skin grafting from autologous skin. Although very effective, these skin substitutes are usually expensive, have poor adhesion properties, are prone to infection, or depend on healthy skin donor availability. In addition, most of the currently clinically used skin substitutes are solely composed of dermal fibroblasts and keratinocytes, and do not promote vascularization. Lack of proper vascularization of the skin grafts and the lack of integration with the recipient tissue is associated with necrosis and graft loss. This generated a high demand for the development of 3D skin tissue for disease studies, drug screening, and potential clinical use [141].

Recently, studies that aimed to induce vascularization in 3D printed skin tissues were designed with the goal to overcome graft necrosis. 

Lee et al. used a collagen hydrogel as scaffold material to print dermal and epidermal compartments of skin tissue with human keratinocytes (HaCaT) and human fibroblasts (HFF-1) layer-by-layer [141]. Printed skin constructs were submerged and cultured in media, followed by culture at the air-liquid interface for additional two weeks to promote stratification. The overall shape, structure and physical dimensions of the printed 3D skin constructs were representative of in-vivo human skin, as demonstrated by H&E and immunofluorescent staining [141]. Although most of the current clinically used skin substitutes are composed of dermal fibroblasts and keratinocytes, the lack of proper vascularization would result to the skin substitutes fail to integration with the recipient tissue and cause the skin grafts necrosis ultimately. Therefore, several groups started to include endothelial cells for vascularization induction in 3D printed skin. 

For example, Yanez et al. fabricated an implantable skin patch composed of three layers: a bi-layered skin structure with a printed vascular layer in the middle [142]. The skin bilayer was composed of a collagen gel loaded with neonatal human dermal fibroblast (NHDF) and a collagen gel with neonatal human epidermal keratinocyte (NHEK). For the printed vascular layer, a bioink composed of fibrin and human dermal microvascular endothelial cells (HMVEC) was used [142]. After 24 h of culture, the printed skin grafts were transplanted to the back of nude mice with full-thickness skin lesions. Uncovered lesions, or full-thickness lesions covered with commercially available skin graft (Apligraf^®^) that contains living epidermis and dermis, were used as the controls. Compared to the controls, the microvascularized skin grafts accelerated wound healing and improved wound contraction by 10%. Functional microvessels, generated from printed HMVEC, were observed in the neoskin after grafting [142]. In a different study, Baltazar et al. printed a 3D multilayered dermal layer of human dermal fibroblasts, endothelial cells, pericytes incorporated in collagen bioink and an epidermal layer with keratinocytes bioink. Endothelial cells in the dermal layer self-assembled into interconnected microvascular networks, demonstrating that the incorporation of pericytes in the dermis stabilizes microvessels and promotes epidermal keratinocyte differentiation [143]. Kim et al. used a 3D cell printing approach to construct a perfusable vascularized human skin tissue composed of epidermis, dermis, and hypodermis [144]. First, pre-adipocyte-loaded adipose fibrinogen hybrid bioink was extruded to create a hypodermal compartment. Then, a gelatin hydrogel containing HUVEC-thrombin was printed in the cylindrical form to generate the vascular channel of the hypodermis. This vascular structure was subsequently covered with a dermal layer consisting of human dermal fibroblast (HDF) suspended in skin specific fibrinogen. After extrusion of the gelation and attachment of HUVECs to the apical lumen of the vascular channel [144], primary human epidermal keratinocytes were printed on the dermal compartment and cultured in the air-liquid interface for epidermal stratification. This printed skin tissue resembled a high similarity with native skin and could potentially be used as a platform for drug screening, cosmetic testing, and disease modeling [144].

Recently, Liu et al. printed 3D matrices of pore structures in different diameters with gelatin hydrogels that contained epithelial progenitors isolated from dorsal skin of E12.5 mice embryos and plantar dermis components. After culturing in sweat gland cell medium, self-organized sweat gland morphogenesis was observed in the 300μm printed constructs [145]. Later they seeded hair follicle spheroids on the 3D printed sweat gland scaffold and found that the interaction between hair follicle spheroids and sweat gland scaffold promoted differentiation of the hair follicles and sweat glands [146]. These results suggest that the use of 3D bioprinting to generate complexity skin tissue including melanocytes and appendages, such as hair follicles and sweat glands could potentially be important for clinical applications of wound treatment.

### 5.5. Kidney

In recent years, many studies aimed to engineer 3D renal tissue for nephrotoxicity prediction, drug screening, and disease modeling.

Homan et al. used a bioprinting method to create 3D convoluted renal proximal tubule (PT) structures on a perfusable chip [147]. To fabricate the 3D PT construct, a fugitive ink was printed on gelatin-fibrin hydrogel and following encase the printed structure by additional hydrogel. After removing the fugitive ink, immortalized human proximal tubule epithelial cells (PTEC) were perfused into the open lumen of the printed structure and PTEC would grow confluent within the tubule after 3 weeks [147]. The mature PT structure was observed in forming a polarized epithelial with columnar cell morphology and revealed the presence of primary cilia in apical surface. Moreover, the improved albumin uptake was also showed in the 3D PT construct compared to PTEC cultured in 2D monolayer. They also demonstrated the potential application for drug toxicity testing by assessing CysA-induced the damage in cell-cell junction by measuring the diffusional permeability of FITC-dextran in the 3D PT constructs [147]. To investigate renal absorption, Lin et al. printed a 3D vascularized proximal tubule model (3D VasPT) that consisted of parallel tubular structures lined with either epithelium or endothelium, in a closed-loop perfusion system [148]. After the 3D VasPT architectures were printed, human proximal tubule epithelial cells (PETC) and glomerular microvascular endothelial cells (GMEC) were seeded in the lumen of the PT and of the vascular channel, respectively, both exhibiting mature phenotypes [148]. Lin et al. modified the composition of gelatin and fibrin in the hydrogel to promote PTEC confluency and demonstrated vectorial transport between the adjacent channels. Additionally, the 3D VasPT exhibited active albumin uptake and glucose reabsorption. By circulating a perfusate with a high glucose level through the 3D VasPT system, they were able to observe the effects of hyperglycemia on kidney cell function, providing a potentially valuable tool to gain insights for treatment of diabetic vascular diseases [148]. Recently, Singh et al. developed a decellularized kidney extracellular matrix (KdECM) and alginate hybrid bioinks for vascularized renal PT bioprinting with the aim to recapitulate the renal 3D microenvironment and to optimize cell functionality and retain the lumen predefined by cells [149]. The printed KdECM PT tubules exhibited a lower level of kidney damage marker KIM-1, suggesting that KdECM bioink significantly improved renal cell function and exerted an anti-fibrotic effect. Toward the realization of a 3D vascularized renal proximal tubule-on-a-chip, PT and blood vessel were printed in coaxial channels using a coaxial nozzle that was connected to a perfusion pump. After perfusion, PTEC and HUVEC lumens exhibited vectorial albumin transport [149]. They transplanted the printed vascularized renal proximal tubules under the kidney capsule of a NOD-SCID mouse model with unilateral ureteral obstruction, and demonstrated long-term survival of the PTEC tubules, suggesting that the printed vascularization supported the maintenance of the graft survival [149]. 

**Table 4:** Applications of the 3D Bioprinting vascularized tissues.

Organs	Cells	Printing Methods	Applications	References
Cardiovascular system	HUVECs, HASMC, HNDFB	Needle array printing system	Tissue regeneration	[43]
	miPSC-DC, HUVECs	Extrusion-based bioprinting	Tissue regeneration	[67]
	hiPSC-DC, hCF	Scaffold-free bioprinting	Disease modeling	[125]
	hiPSC-CS, hNDF	SWIFT (sacrificial writing into functional tissue) method	In vitro model Drug treatment screening	[126]
	C2C12, hESC-CM, CF	extrusion-based bioprinting	in vitro model	[127]
Liver	HepG2/C3A cells	Extrusion-based bioprinting	Drug toxicity testing	[131]
	hHep, hepatic stellate cells, HUVECs	Extrusion-based bioprinting	Drug toxicity testing.	[132]
	HUVECs, hMSCs, hHep	Needle array printing system	Tissue regeneration	[134]
	hHep, HUVECs, NHDF	Sacrificial bioprinting	Tissue regeneration	[135]
	rHep, HUVEC, NHDF	Stereolithography	Tissue regeneration	[136]
Brain	rPeri, Astro, NSCs, bEnd.3	Sacrificial bioprinting	In vitro model Drug treatment screening	[137]
	hGlio, hAstro, microglia, HUVEC, PHMBP	Sacrificial bioprinting	In vitro 3D tumor model Drug treatment screening	[140]
Skin	nHDF, nHEK, HMVEC	Extrusion-based bioprinting	Tissue regeneration	[142]
	hDF, Endo, Peri, Kera	Extrusion-based bioprinting	Tissue regeneration	[143]
	hDF, hEK,, HUVECs, pre-adi	Extrusion-based bioprinting Sacrificial bioprinting	in vitro model Drug treatment screening	[144]
kidney	PTEC	Sacrificial bioprinting	Disease modeling Drug toxicity testing	[147]
	PTEC, GMEC	Sacrificial bioprinting	Disease modeling Drug treatment screening	[148]
	PTEC, GMEC	Extrusion-based bioprinting with a core fugitive material	Tissue regeneration	[149]

HUVEC = Human umbilical vein endothelial cells; HASMC = human aortic smooth muscle cells; HNDFB = human normal dermal fibroblasts; hiPSC-DC = hiPSC-derived cardiomyocytes; hCF = human cardiac fibroblasts; hiPSC-CS = hiPSC-derived cardiac spheroids; hNDF = human neonatal dermal fibroblasts; C2C12 (Mouse skeletal muscle cells); hESC-CM = hESC-derived cardiomyocytes; CF = cardiac fibroblasts; hHep = primary human Hepatocytes; NHDF = normal human dermal fibroblasts; rHep = primary rat hepatocytes; rPeri = Primary rat Pericytes; bEnd.3 (brain endothelial cell line); hGlio = Human glioblastoma cells; hAstro = Human astrocytes; PHMBP = primary human microvascular brain pericytes; nHDF = neonatal human dermal fibroblasts; nHEK = neonatal human epidermal keratinocytes; HMVEC = human dermal microvascular endothelial cells; hDF = human dermal fibroblasts; endo = endothelial cells; Peri = Pericytes; Kera = Keratinocytes; hEK = human epidermal keratinocytes; pre-Adi = pre-adipocytes, PTEC = human proximal tubule epithelial cells; GMEC = glomerular microvascular endothelial cells.

## 6. Conclusions, Limitations, and Future Directions

Developing a mature and well-perfused vascular network inside a growing tissue is critical to the success of tissue and organ regeneration. Bioprinting offers remarkable benefits for vascular network formation, either as a vascular tissue or blood vessel-like structures. As a result, the printing of vascular structures has made tremendous progress to date. To make bioprinting effective, tissue engineering, material science, and medical researchers have created a range of unique 3D printing processes and biocompatible materials to optimize printing and mechanical properties, biodegradability, biocompatibility, and low cytotoxicity. Due to its practicality, a range of accessible printing technologies, precision, and control over the printing process, 3D printing has piqued the interest of many researchers as a non-substitutable approach for the permanent creation of vascular networks. The technological advances of 3D bio-printing the recent years have made it feasible to print cell aggregates, tissue strands, or cell and growth factor-loaded biomaterials layer-by-layer according to a predefined shape created by CAD software.

Despite advances in 3D bioprinting technology, fabricating vascular tissue and blood vessels as a therapeutic replacement still harbors some problems. To date, although several bioprinted products are commercially available, they aim to replace traditional pharmacological testing and are not design the replace human organs. To our knowledge, only five clinical trials about bioprinting products have been registered on the clinicaltrials.gov website. Only two includes structures bioprinted with the intended use for human implantation, and none have a clear focus on vascularized tissue or vascularization. It is thus clear that, despite what popular scientific journals claim, there is still challenges that need to be overcome before bioprinted vascularized tissues reach clinical translations. These challenges can be grouped into three fields: (i) physical characteristic of liquids and small volumes limiting the generation of small blood vessels, (ii) technological limitations that inhibit the linear progression to increase printing resolution, (iii) development of protocols for the differentiation of patient derived iPSC to endothelial progenitors. 

The physical characteristics of liquids, hydrogels and cells that are deposited to generate a 3D printed object are setting the framework for technical solutions. Best printing results can be obtained with highly viscous hydrogels that show rapid polymerization behavior. Increased viscosity requires high pressure at the printing nozzle to overcome clogging. The pressure on liquids or gels in extrusion printing, is limited by the maximum shear force that can be applied without reducing the viability of the cellular components of the bioink. A liquid’s behavior in a droplet-based bioprinting nozzle influences the smallest possible droplet size and subsequently limits the smallest vessel size that can be printed. 

Technological limitations apply to every level of bioprinting. First is the production of structures that anatomically mimic the layered structures of blood vessels with specific cells and proteins to permit physiological characteristics. Improvements in printing resolution and precision are necessary to produce tubular structures that cover vessel with diameters ranging from centimeters to micrometers, including the precise cell organization and thickness. Resolution and precise control over of the correct placement of biomaterials, ECs, vascular SMCs, and growth factors pose a challenge for printing vascularized tissues with complex hierarchical vascular networks (e.g., hepatic or renal tissues) that suit the host tissues. For example, at the time we are writing this review, there is no technique available to print the tree-like hierarchical branching structure of blood vessels in a single process, and the printing of capillaries has not been demonstrated at all.

The generation of patient-derived cells via iPS seems a viable option to generate cellular material for bioprinting approaches in regenerative medicine. However, the isolation of cells and the subsequent differentiation to tissue-specific lineages is not a simple task. For example, the generation of patient-derived endothelial progenitors from iPS cells is a process that takes a significant time and involves rate-limiting steps, which is further challenged by the high number of cells necessary for bioprinting. Robust differentiation protocols and potentially automated processing will be necessary to ensure homogenous cell populations.

Progress is necessary to formulate biomaterials that enable the generation of scaffolds that better support cell growth. In addition, these materials must exhibit suitable mechanical properties to maintain the tissue structure during cell growth after biomaterial biodegradation and prevent collapsing in a perfusion environment. Because the choice of printing materials is essential for the culture of specific cell types, efforts have to be made to produce a biomaterial that can support the production of tissues with different cell types. Another area that requires improvement is the development of materials that are compatible with existing printing processes in terms of printability and speed, as these factors influence the form fidelity of printed structures. 

## Figures and Tables

**Table 1:** Advantages and Disadvantages of the Different 3D Bioprinting Methods.

Materials Application	Printer Style	Advantages	Disadvantages
Extrusion-based	Bioplotting	wide range of bioinks	slow printing process
	Fusion deposition modelling	wide range of bioinks	limited resolution only hydrogels slow
Laser-assisted	Stereolithography	high resolution possible	UV damage to cells small range of bioinks
Droplet-based	Inkjet	gentle to printed cells fast printing affordable	limitations of cell density low resolution

## Data Availability

Not applicable.
